# Tissue-protective activity of selenomethionine and D-panthetine in B16 melanoma-bearing mice under doxorubicin treatment is not connected with their ROS scavenging potential

**DOI:** 10.3325/cmj.2017.58.171

**Published:** 2017-04

**Authors:** Rostyslav R. Panchuk, Nadia R. Skorokhyd, Yuliya S. Kozak, Liliya V. Lehka, Andrey G. Moiseenok, Rostyslav S. Stoika

**Affiliations:** 1 Department of Regulation of Cell Proliferation and Apoptosis, Institute of Cell Biolog,y NAS of Ukraine, Lviv, Ukraine; 2 Department of Biochemistry, Biological Faculty, Ivan Franko Lviv National University, Lviv, Ukraine; 3 Department of Vitaminology and Nutraceuticals, Institute of Biochemistry of Biologically Active Compounds, Leninskoho, Belarus

## Abstract

**Aim:**

To evaluate molecular mechanisms of tissue-protective effects of antioxidants selenomethionine (SeMet) and D-pantethine (D-Pt) applied in combination with doxorubicin (Dx) in B16 melanoma-bearing-mice.

**Methods:**

Impact of the chemotherapy scheme on a survival of tumor-bearing animals, general nephro- and hepatotoxicity, blood cell profile *in vivo,* and ROS content in B16 melanoma cells *in vitro* was compared with the action of Dx applied alone. Nephrotoxicity of the drugs was evaluated by measuring creatinine indicator assay, hepatotoxicity was studied by measuring the activity of ALT/AST enzymes, and myelotoxicity was assessed by light microscopic analysis of blood smears. Changes in ROS content in B16 melanoma cells under Dx, SeMet, and D-Pt action *in vitro* were measured by incubation with fluorescent dyes dihydrodichlorofluoresceindiacetate (DCFDA, H_2_O_2_-specific) and dihydroethidium (DHE, O_2_^-^-specific), and further analysis at FL1 (DCFDA) or FL2 channels (DHE) of FACScan flow cytometer. The impact of aforementioned compounds on functional status of mitochondria was measured by Rhodamine 123 assay and further analysis at FL1 channel of FACScan flow cytometer.

**Results:**

Selenomethionine (1200 µg/kg) and D-pantethine (500 mg/kg) in combination with Dx (10 mg/kg) significantly reduced tumor-induced neutrophilia, lymphocytopenia, and leukocytosis in comparison to Dx treatment alone. Moreover, SeMet and D-Pt decreased several side effects of Dx, namely an elevated creatinine level in blood and monocytosis, thus normalizing health conditions of B16 melanoma-bearing animals.

**Conclusions:**

Our results showed that antioxidants selenomethionine and D-pantethine possess significant nephroprotective and myeloprotective activity toward Dx action on murine B16 melanoma *in vivo*, but fail to boost a survival of B16 melanoma-bearing animals. The observed cytoprotective effects of studied antioxidants are not directly connected with their ROS scavenging.

Golden chemotherapy standards (eg, anthracycline antibiotics, vinca alkaloids, inorganic platinum compounds) have been used for decades for treatment of advanced cancers (1). Despite different mechanisms of action (inhibition of DNA topoisomerase II, blockage of tubulin polymerization, DNA intercalation), their negative side effects are similar and include myelosuppression, nausea, mucositis, alopecia, renal damage, thus, leading to significant worsening of health conditions of cancer patients (2). Doxorubicin (Dx) is one of the most widely used chemotherapeutics possessing broad spectrum of anticancer activity. Its therapeutic potential is realized through various pathways, including DNA intercalation, DNA topoisomerase II inhibition, and induction of the oxidative stress in target cells (3). Numerous data indicate that Dx-mediated ROS production is the leading reason of an acute myelotoxicity and nephrotoxicity of this drug (4,5). However, the most dangerous consequence of Dx-induced oxidative stress is a delayed cardiac failure found in 18% cancer patients who received more than 551 mg/m^2^ dose of this drug (6). Thus, novel approaches should be developed in order to enhance selectivity of action of the chemotherapies and diminish their toxic effect toward normal tissues of the organism.

Dietary antioxidant supplementations might be promising candidates for this role, though their use for treatment of various diseases remains a controversial topic for last decades. Initially popularized in mid-1970s by Nobel laureate Linus Pauling, antioxidants are now used by a majority of cancer patients, since it is considered that they decrease potential harmful effects of the chemotherapy and, thus, improve a quality of patients’ life (7). This tendency is constantly growing even despite the results of numerous clinical trials showing that the antioxidants had no real impact on a survival of cancer patients, or some of them (vitamins A and E) even worsened their prognosis (8,9).

In the above mentioned trials, selenium was found to be the only dietary supplement demonstrating the anti-tumorigenic activity and, thus, it was considered to be a promising candidate for further application in chemotherapy (8). This might be of great importance for the regimens including Dx, as its combination with the antioxidant (eg, selenium) should improve therapeutic action of Dx and also lower drug-induced oxidative stress.

Up to now, there is little information regarding therapeutic efficiency of the organo-selenium compounds in cancer treatment. The main aim of this work was to study in more detail the mechanisms of tissue-protective and therapeutic activity *in vivo* of selenomethionine in comparison with D-pantethine – vitamin B_5_ precursor. Previously, we have shown that both SeMet and D-Pt decreased the oxidative stress in tissues of healthy rats treated with the anthracycline antibiotic - doxorubicin (Dx) (10). Those studies were repeated on mice bearing NK/Ly lymphoma and revealed that both antioxidants used in a combination with Dx not only decreased the lymphopenia and monocytosis caused by Dx, but also led to an increase in animal survival time comparing to Dx treatment (11).

Since leukemias and lymphomas are considered to be the most sensitive tumors toward Dx action, the effect of using antioxidant supplementations can be hardly noticeable on the background of strong therapeutic effect of Dx alone. In order to see a more pronounced effect of the combined action of Dx and antioxidants, we addressed murine B16 melanoma that is considered to be one of the most resistant tumors regarding Dx action (12,13). In the present paper, therapeutic efficiency of the antioxidants in mice bearing B16 melanoma was studied, in particular, the impact of proposed regimen on animal survival, blood profile, hepatotoxicity, and nephrotoxicity. Additionally, identification of potential ROS-scavenging activity of SeMet and D-Pt in B16 melanoma cell line under Dx treatment in vitro was dissected by analysis of their influence on ROS production, namely, hydrogen peroxide and superoxide anions, and functional status of mitochondria.

## Materials and methods

### Materials

Seleno-L-methionine (≥98% (TLC)) and D-pantethine were purchased from Sigma (St. Louis, MO), and doxorubicin hydrochloride was obtained from Pfizer (New York, NY). Antioxidants were dissolved in sterile 0.9% sodium chloride solution prior to *per os* treatment of animals or addition to cell culture.

### Cell culture and treatments

Murine B16 melanoma cells were kindly provided by Prof. Walter Berger (cell culture collection at Vienna Medical University, Institute of Cancer Research). Cells were cultured in RPMI medium supplemented with 10% fetal calf serum (Sigma-Aldrich, St. Louis, USA), 50 µg/mL streptomycin (Sigma-Aldrich, St. Louis, USA), 50 units/mL penicillin (Sigma-Aldrich, St. Louis, USA) in 5% CO_2_-containing humidified atmosphere at 37°C. For experiments cells (2*10^5^ per well) were seeded into 24-well tissue culture plates (Greiner Bio-one, Germany). Short-term (24 h) cytotoxic effect of antitumor drugs was studied under the Evolution 300 Trino microscope (Delta Optical, Poland) after cell staining with Trypan blue (0.1%).

### Flow cytometric assays

Rhodamine 123 (Rh123) accumulation assay was performed as previously described (14). Briefly, 2x10^5^ B16 melanoma cells were incubated with studied compounds for the indicated time points at 37°C. 1 hour before termination of incubation, Rh123 (0.25 mg/mL) was added and cell-bound fluorescence was collected through a 530/30 nm band-pass filter (FL1 channel) of FACScan flow cytometer (BD Biosciences, San Jose, CA). ROS content in cells was measured by incubating control or drug-treated cells with fluorescent dyes dihydrodichlorofluoresceindiacetate (DCFDA, detecting mainly H_2_O_2_) or dihydroethidium (DHE, superoxide-specific) in concentrations of 10 μM at 37°C for 30 min. After incubation with the dyes cells were washed with PBS and immediately analyzed at FL1 (DCFDA) or FL2 channel (DHE) of FACScan flow cytometer (BD Biosciences, San Jose, CA).

### Animal studies

**Figure 1:**
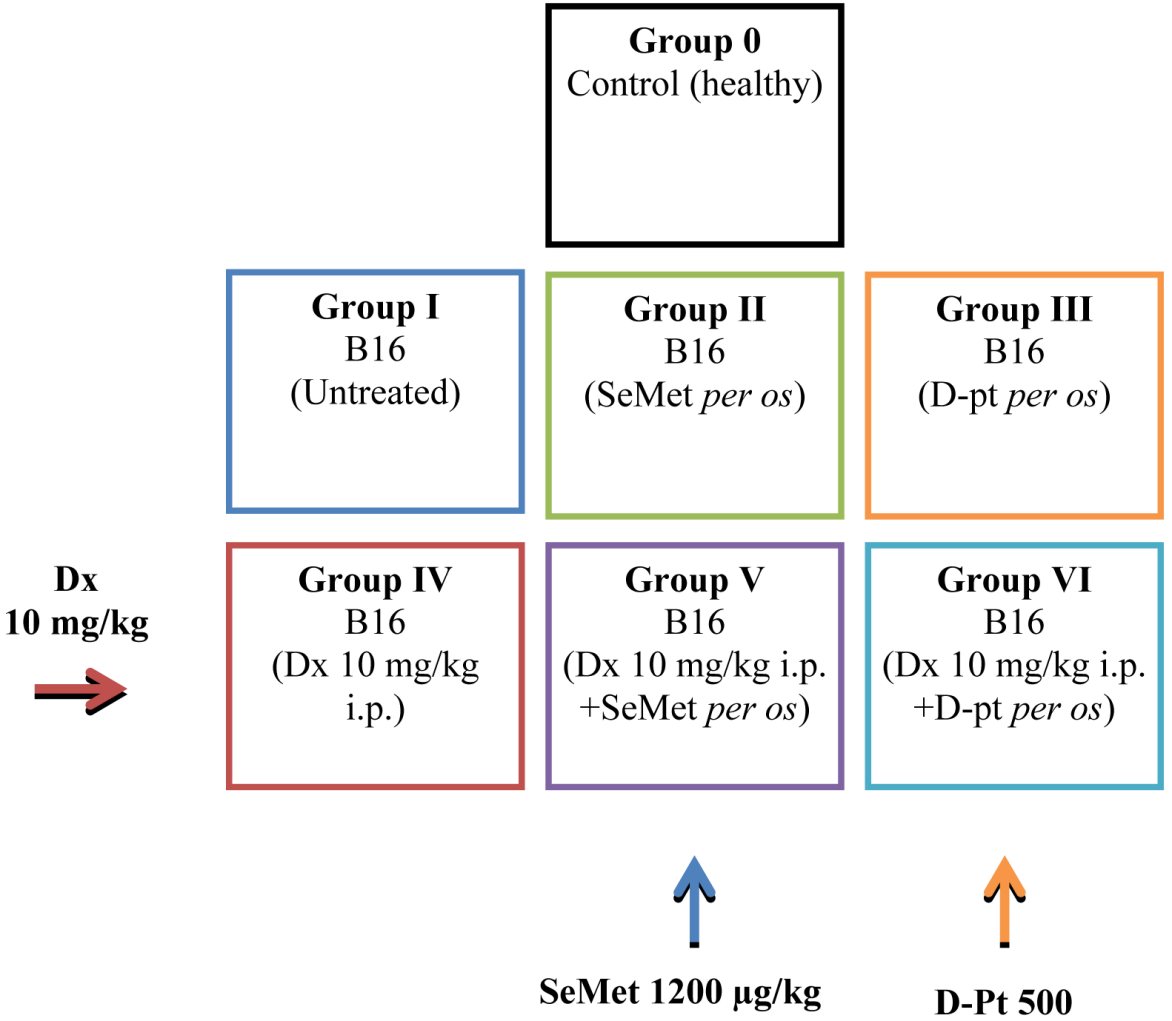
Groups of animals in the study.

Studies of the biological activity of selenomethionine, D-pantethine, and doxorubicin were conducted in 2016 at the animal facility of the Institute of Cell Biology, NAS of Ukraine (Lviv, Ukraine). 42 adult male С57/Bl6 mice with 25-28 g weight were kept under standard vivarium conditions with constant access to the full feed and drinking water. Tumor inoculation was conducted by a subcutaneous injection of B16F10 cell suspension (10^6^ cells per animal) diluted with sterile phosphate buffered saline (PBS) in right rear paw of mice. The viability and number of cells stained with 0.1% Trypan blue were checked by cell counting in the hemocytometric chamber. The vitality of melanoma cells used for transplantation was not less than 98%. Animals were divided into 7 groups with 6 mice in each group (Figure 1). Blood sampling for biochemical and cytomorphological studies was done at 20^th^ day after tumor inoculation (and the 10^th^ day after chemotherapy start).

Mice from experimental groups were administered selenomethionine (120 µg/kg, cumulative dose 1200 µg/kg) (groups 2, 5) or D-pantethine (50 mg/kg, cumulative dose 500 mg/kg) (groups 3, 6) *per os* every second day, starting from 10^th^ till 30^th^ day of tumor inoculation. Mice of zero group (healthy) and first group (B16 melanoma, untreated) received simultaneously the equivalent volume of 0.9% sodium chloride solution in a similar mode. Doxorubicin (1 mg/kg, cumulative dose 10 mg/kg) was injected i.p. every second day starting from the 10th to the 30th day of tumor inoculation to the animals of groups 4-6. Treatment of animals with antioxidants in groups 5-6 took place 1 h before Dx injection. The chemotherapy scheme was developed, based on NCI recommendations (15) and our previous results (10,11).

All *in vivo* experiments were conducted in accordance with the international principles of the European Convention for protection of vertebrate animals under a control of the Bio-Ethics Committee of the above mentioned institution (Protocol N 4/2016 from 5.06.2016 of the BioEthics Committee at the Institute of Cell Biology, NAS of Ukraine).

### Myelotoxicity studies

For blood sampling, amputation of a small part of mouse tail was done, pumping of ~ 50 µL of blood in a test tube, followed by immediate disinfection of a wound with 70% alcohol. For counting of red blood cells, 5 µL of blood were dissolved in 5 ml of isotonic NaCl solution (1:1000 dilution), while for leukocyte 5 µL of blood was dissolved in 95 µL of 3% acetic acid solution (1:20 dilution). Erythrocytes were counted in 5 big squares (divided into 16 small ones) of the hemocytometric chamber, while leukocytes were counted in 100 big squares, grouped by 4, under the Evolution 300 Trino microscope (Delta Optical, Mińsk Mazowiecki, Poland). The number of erythrocytes and leukocytes was counted using standard formulas, described in (16).

For blood smear preparation, 3 µL of blood was put at the edge of a slide, and then spread for 1.5 cm using another narrow polished slide, placed at a 45° angle. The obtained smears were dried at room temperature, fixed with absolute methanol, and then rehydrated by subsequent washing in ethanol solutions with decreasing concentrations (96%, 75%, 50%, 25%, and 12.5%). Finally, the smears were washed with distilled water, stained with Giemsa dye using standard protocol and air-dried, after which they were ready for leukogram analysis.

Counting of leukocytes was performed under Evolution 300 Trino microscope (Delta Optical, Mińsk Mazowiecki, Poland) on 90 × oil immersion objective. Cell counting was always done using the same system – half of cell population was counted in the upper half part of the smear, and the other half was counted on the lower part of the smear. The percentage of certain types of white blood cells in each smear was determined after counting of at least 300 cells. The obtained values (due to differences of absolute numbers of cells in each counted smear) were normalized to 100%, and percent values of each leukocyte fraction were calculated as described in (17).

### Hepatotoxicity studies

For measuring the aspartate aminotransferase activity, 10 µL of blood serum was mixed with 100 µL of substrate solution (2 mM α-ketoglutaric acid; 0.2 M D,L-aspartate in 0.1 M phosphate buffer pH 7.4), while in control tube 10 µL of distilled water were added instead of serum. The tubes were placed for 60 min at 37°C, and then 100 µL of 1 mM solution of 2,4-dinitrophenylhydrazine was added to the samples and left for 20°C at room temperature. After it 1 ml of 0.4 M sodium hydroxide solution was added to each sample for extra 10 min, and the optical density of the samples was measured using Thermo Spectronic spectrophotometer (Helios, Great Britain) at 540 nm wavelength. For measuring the alanine aminotransferase activity, procedure was identical except substrate solution (2 mM α-ketoglutaric acid; 0.2 M D,L-alanine in phosphate buffer pH 7.4).

### Nephrotoxicity studies

Creatinine level in blood serum of experimental animals was measure spectrophotometrically using Popper method based on Jaffe reaction (18). Briefly, blood serum samples were diluted 1:20 in working reagent solution (0.75M NaOH and saturated picric acid, mixed 1:1), and their optical density was measured at Thermo Spectronic spectrophotometer (Helios, Great Britain) at 510 nm wavelength after 30 (E_1_) and 90 sec (E_2_) following sample addition to working reagent solution. The obtained results were compared to etalon (creatinine solution, 440 µM) and the final creatinine content in blood serum samples was calculated using following formula.



All experiments were performed in triplicate and repeated 3 times. Statistical analysis of data was conducted in GraphPad Prisms Software (GraphPad Software, Inc) using Student’s *t* test. Statistical significance was set at *P* ≤ 0.05.

## Results

The adverse effects of many anticancer drugs are the main drawbacks that accompany their use. Thus, application of specific non-drug agents that reverse these effects can significantly improve the treatment action of traditional anticancer drugs. Previously, we have shown that dietary compounds selenomethionine and D-pantethine partially decreased hepatotoxicity and myelotoxicity of doxorubicin in NK/Ly lymphoma-bearing mice (11). We suggested that tissue-protective effect of these antioxidants might be explained either by their direct scavenging of Dx-induced ROS or by protecting mitochondria that are considered to be the major source of cell-produced ROS (19) during damaging effect of Dx. In order to confirm this hypothesis, *in vitro* studies of the combined action of Dx and antioxidants were performed. B16 murine melanoma was selected for several reasons: a) it is internally resistant to Dx action, thus, any enhancement of its cytotoxic activity toward tumor cells by the antioxidants will be well seen. This is in contrast to other cellular models, which are very sensitive to Dx action, and a moderate effect of other compounds can be missed; b) B16 melanoma can be used both *in vitro* and *in vivo* on C57/Bl6 mice, allowing immediate verification of *in vitro* data using the same animal tumor model.

Such a combined *in vitro-in vivo* approach should be of great importance for identification of potential clinical markers of drug-induced oxidative stress and mitochondrial dysfunction, as well as their modulation by studied antioxidants.

### Selenomethionine and D-pantethine do not protect B16 melanoma cells from Doxorubicin-induced mitochondrial damage and have a little modulatory action on the level of superoxide anions increased under Doxorubicin action

**Figure 2:**
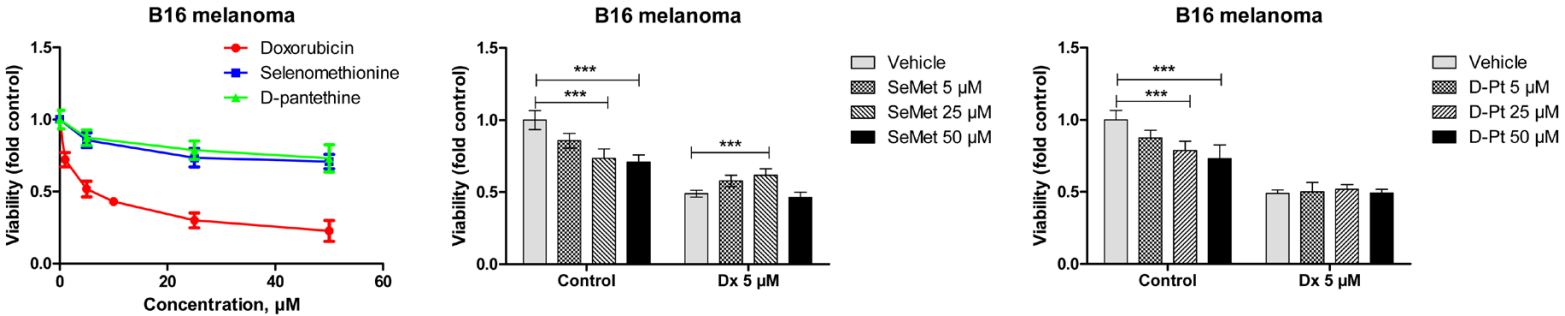
*In vitro* study of cytotoxic activities of SeMet, D-Pt and Dx and their combined treatment on B16 melanoma cells. Viability of Jurkat cells following exposure to the indicated concentrations of Dx in combination with SeMet or D-Pt was analyzed after 24 hours by trypan blue exclusion. The effect of Dx on cell growth was plotted relative to the untreated control. Data given represent the mean±SD of three independent experiments done in triplicates. ***P < 0.001, unpaired *t* test.

We have checked if the observed tissue-protective effects of D-Pt and SeMet can be explained by their ability to scavenge produced toxic reactive oxygen species (ROS). Thus, the impact of these antioxidants on the level of specific ROS (namely, hydrogen peroxide and superoxide anions) was studied in cultured B16 melanoma cells. For selecting optimal (eg, non-toxic) concentrations of SeMet and D-Pt, as well as for evaluation of LC_50_ dose (lethal concentration of drug killing 50% cells) of Dx, Trypan blue cytotoxicity assay was used (Figure 2). One can see that B16 melanoma is characterized by an internal resistance to Dx (LC_50_ 5 µM, and LC_75_ 25 µM). SeMet and D-Pt do not possess cytotoxic action toward B16 cells even used in high doses (50 µM), while in low concentrations (5-25 µM), SeMet partially (by 10%-12%, *P* < 0.001) inhibited cytotoxic action of Dx toward studied tumor cells (Figure 2).

**Figure 3:**
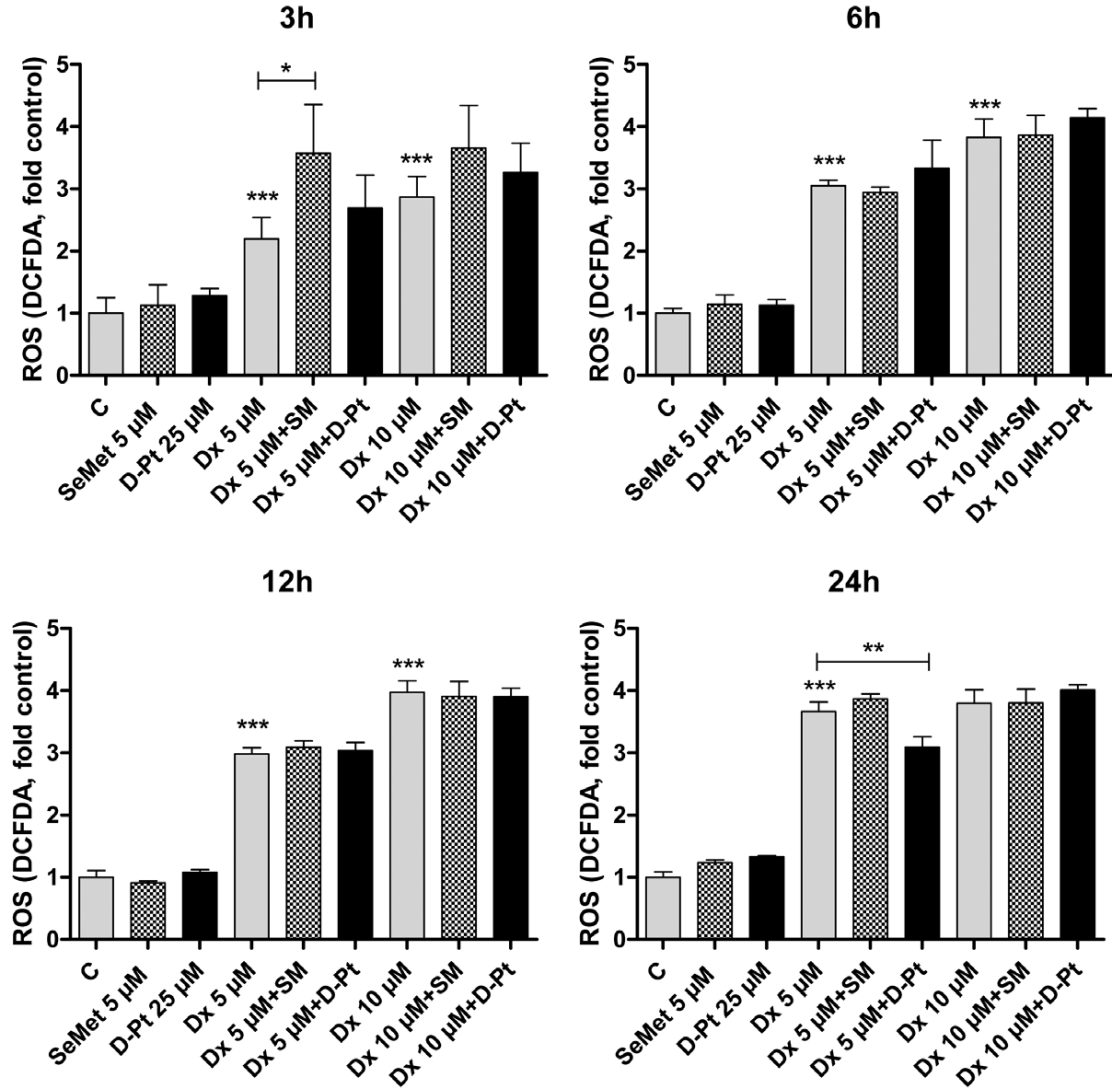
Impact of SeMet and D-Pt on hydrogen peroxide production in B16 melanoma cells under Dx treatment. DCFDA fluorescence of B16 cells indicative for H_2_O_2_ generation was determined by flow cytometry at the indicated time points after addition of Dx to B16 cell culture without and with addition of 5 µM of SeMet and 25 µM of D-Pt. Data are given relative to the untreated control samples and represent the mean±SD of three independent experiments. *P < 0.05 relative to control, **P < 0.01 relative to control, ***P < 0.0001 relative to control, unpaired *t* test. Significance levels indicated directly above bars refer to the comparison with the respective vehicle-treated controls.

**Figure 4:**
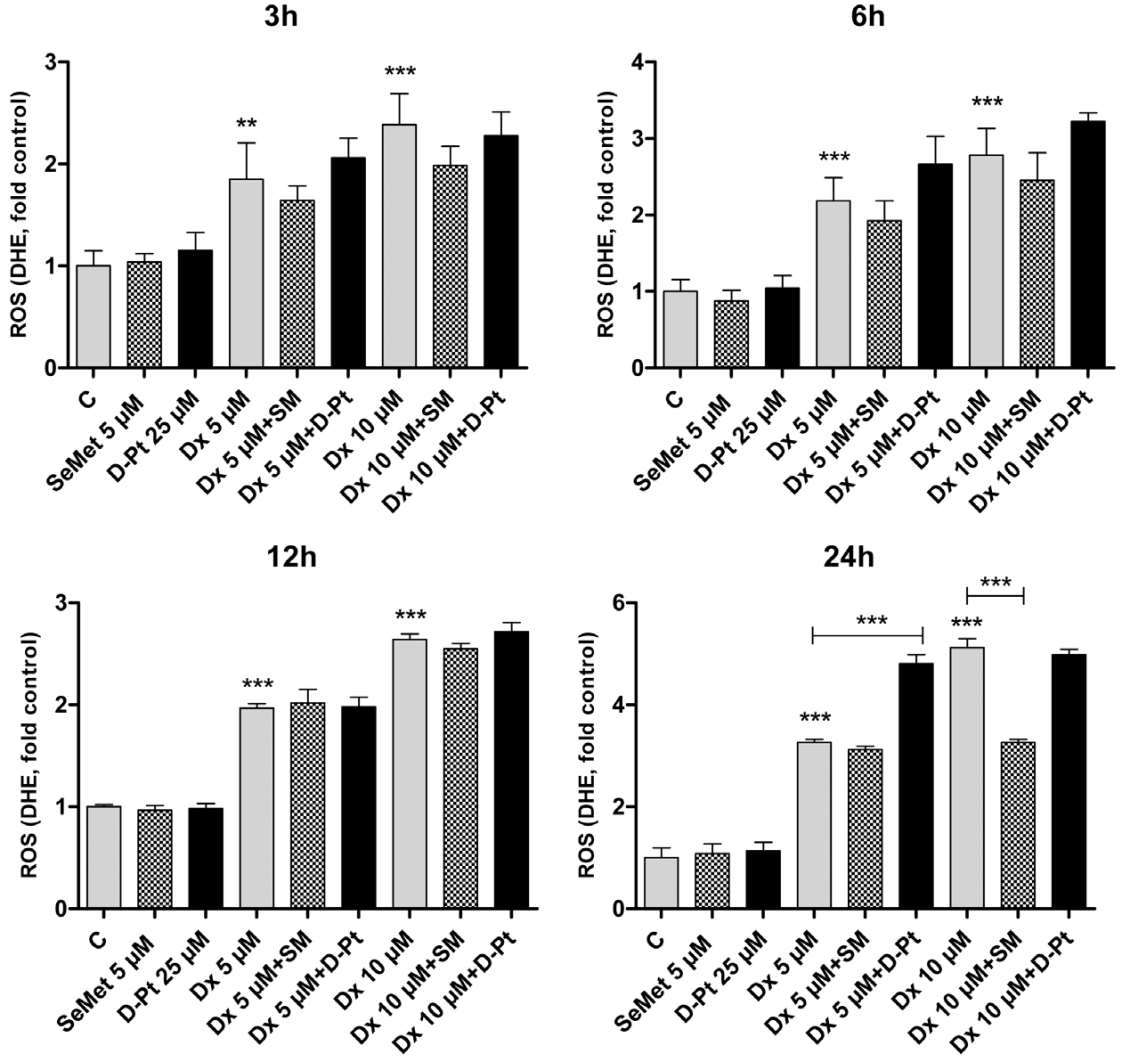
Impact of SeMet and D-Pt on superoxide anions production in B16 melanoma cells under Dx treatment. DHE fluorescence of B16 cells indicative for O_2_^-^ generation was determined by flow cytometry at the indicated time points after addition of Dx to B16 cell cultures without and with addition of 5 µM of SeMet and 25 µM of D-Pt. Data are given relative to the untreated control samples and represent the mean±SD of three independent experiments. *P < 0.05 relative to control, **P < 0.01 relative to control, ***P < 0.0001 relative to control, unpaired *t* test. Significance levels indicated directly above bars refer to the comparison with the respective vehicle-treated controls.

**Figure 5:**
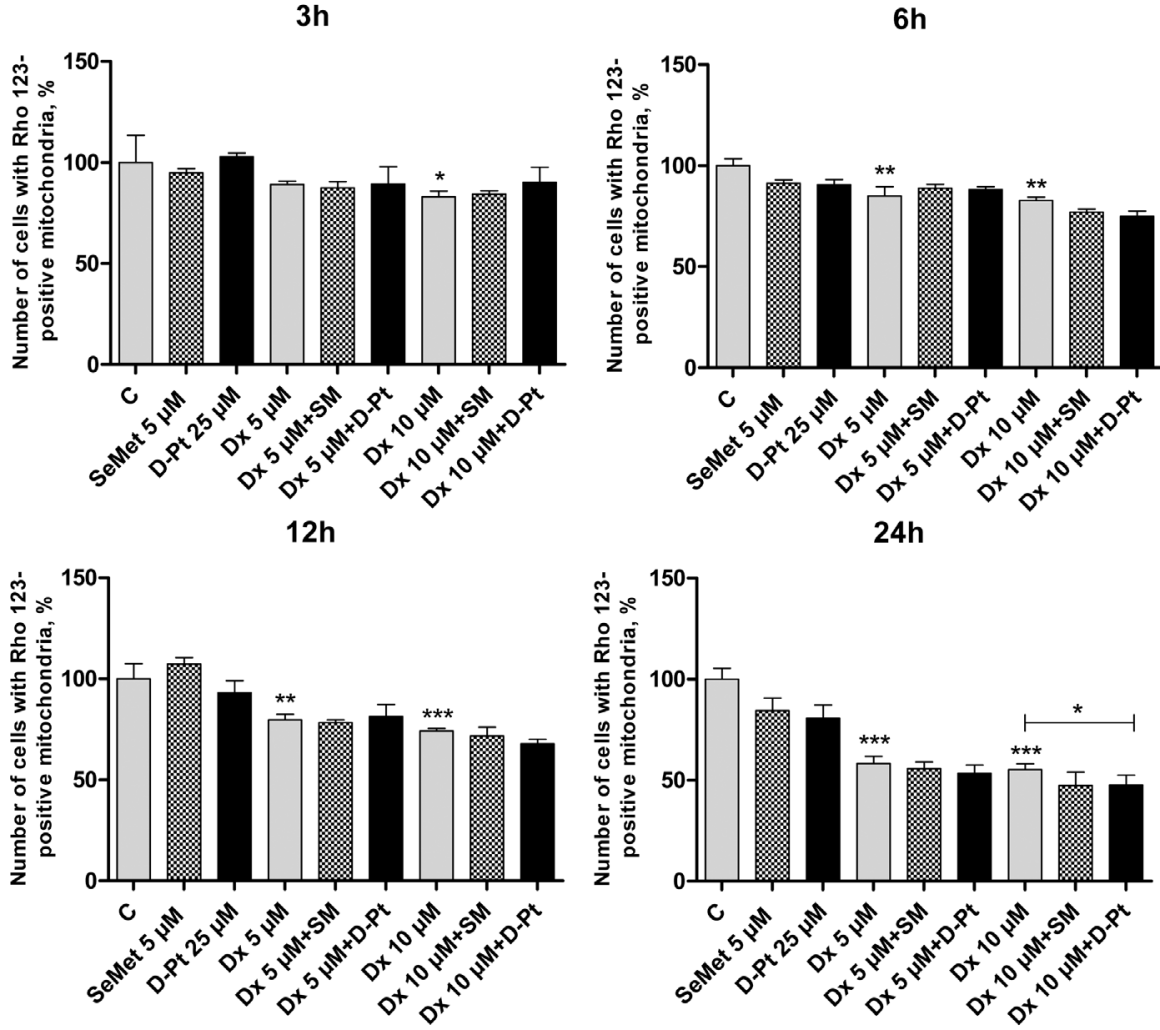
Impact of SeMet and D-Pt on Dx-mediated mitochondrial degradation. Cells were stained with Rho 123, pre-treated for 30 min with NAC, then treated with either vehicle, LC_50_ (5 µM) and LC_75_ doses of Dx (10 µM) and analyzed by flow cytometry. Data depict means and SD of three independent experiments in duplicate. Values are given relatively to the vehicle-treated controls. *P < 0.05, **P < 0.01, ***P < 0.001, unpaired *t* test. Stars directly above the bars indicate differences to the respective vehicle controls.

Cytotoxic effect of Dx toward B16 cells was accompanied by a significant time-dependent increase in the level of both hydrogen peroxide (measured by DCFDA fluorescence assay) and superoxide anions production (measured by DHE assay) (Figures 3 and 4). In particular, H_2_O_2_ concentration increased 2-fold already at 3 h after Dx addition to cell culture, and further enhanced up to 4-fold at 24 h time point (Figure 3). The same fluctuations were observed for Dx-induced O_2_- radicals whose level increased 2-fold at 3 h, also reaching its peak at 24 h time point (Figure 4). These events tightly correlated with mitochondrial damage, measured by Rhodamine 123 accumulation assay (Figure 5). Dx in LC_50_ dose (5 µM) led to disruption of 10% of cellular mitochondria at 3 h, 15% - at 6 h, 20% - at 12 h, while at 24 h time point this number increased to 42%. Thus, Dx-induced mitochondrial damage leads to time-dependent increase of both hydrogen peroxide and superoxide anions production in Dx-treated cells.

Surprisingly, no major ROS scavenging effects were observed at the action of SeMet or D-Pt (Figures 3-5). On the contrary, SeMet (5 µM) increased hydrogen peroxide in 3 h after Dx addition to cultured cells (Figure 3). At later time points, (6 h, 12 h, 24 h), no significant difference in the action of Dx and its combination with SeMet or D-Pt on the level of hydrogen peroxide was observed (Figure 3). D-Pt also had no effects on the level of superoxide anions under Dx treatment, while SeMet partially decreased it at 24 h in case of using high (10 µM) dose of Dx (Figure 4).

Finally, both studied supplements had no effect on functional status of mitochondria in B16 cells damaged by Dx (Figure 5). Thus, SeMet and D-Pt possessed a little cytoprotective impact toward Dx cytotoxic action on the melanoma cells *in vitro*, and had no impact on mitochondrial dysfunction and subsequent oxidative stress induced by Dx in these cells.

### Selenomethionine, in contrast to D-pantethine, causes partial inhibition of growth of B16 melanoma in mice and enhances a therapeutic action of Dx

**Figure 6:**
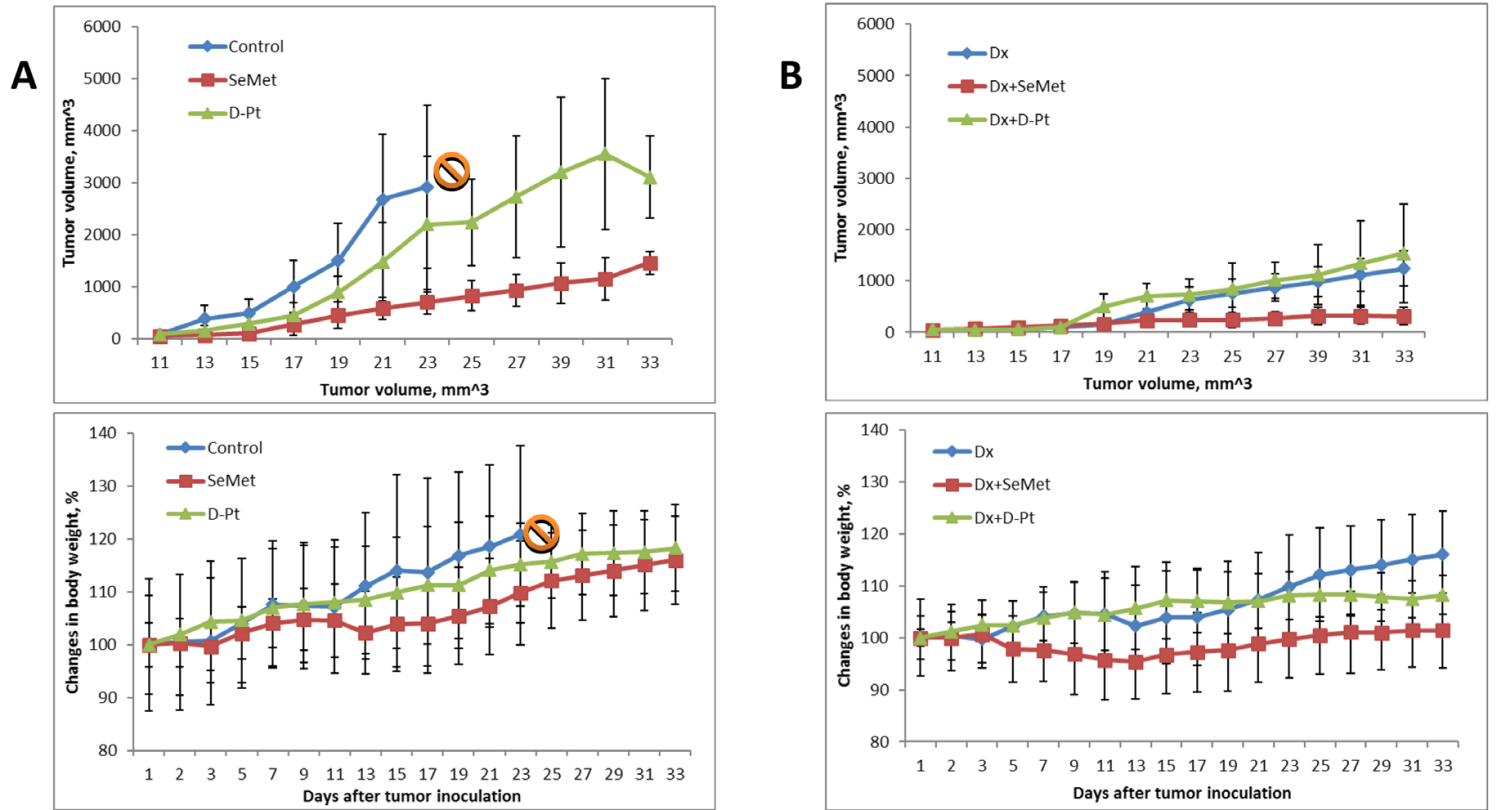
Changes in tumor volume and body mass of animals with B16 melanoma treated with D-Pt and SeMet alone (**A**) or in combination with Dx (**B**). Tumor volume was measured every other day starting from 10^th^ day after melanoma inoculation according to materials and methods section. Animal weight was measured every other day, starting from 1^st^ day of tumor inoculation.

In previous studies (11), we demonstrated that SeMet and D-Pt increased both survival and quality of life on mice bearing NK/Ly lymphoma. In case of B16 melanoma, a direct comparison of animal survival time in different groups was not possible due to specificity of this solid tumor model in which rapid development of large necrotic nodules takes place. Thus, tumor-bearing mice had to be euthanized according to ethical reasons before their death caused by tumor-induced intoxication and cachexia. In particular, animals of control group were euthanized at 22^nd^ day after tumor inoculation when tumor volume reached 3 cm^3^ (Figure 6A). D-Pt possessed a weak tumor-inhibitive action, and the implanted B16 melanoma in D-Pt-treated animals reached its maximum allowable size only at the 33^rd^ day after its inoculation. On the contrary, SeMet alone significantly inhibited B16 melanoma growth whose volume was only 1 cm^3^ at the 33^rd^ day after tumor inoculation, thus, suggesting a major therapeutic effect of this dietary supplement toward B16 melanoma (Figure 6A).

Despite high internal resistance of B16 melanoma to Dx action revealed by us in vitro (Figure 2), it positively responded to Dx therapy in vivo (Figure 6B), and at the 33^rd^ day after tumor inoculation, average size of tumor nodules was less than 900 mm^3^. A combination of Dx and D-Pt had not revealed a cumulative effect on B16 melanoma growth, suggesting little therapeutic importance of D-Pt in this model. On the contrary, a combination of Dx and SeMet possessed a strong synergistic effect on B16 melanoma progression and efficiently inhibited its growth at the 33^rd^ day after tumor inoculation (average tumor volume was 320 mm^3^, *P* < 0.05) (Figure 6B). At the 60^th^ day after B16 melanoma inoculation, these differences between Dx and Dx+SeMet groups were still visible, but not statistically significant due to high variability of sizes and necrotization of tumor nodules. That was the reason for conducting euthanization of tumor-bearing animals for the ethical reasons.

### Selenomethionine and D-pantethine decrease nephrotoxicity and myelosuppressive effects of Dx in mice with B16 melanoma

**Figure 7:**
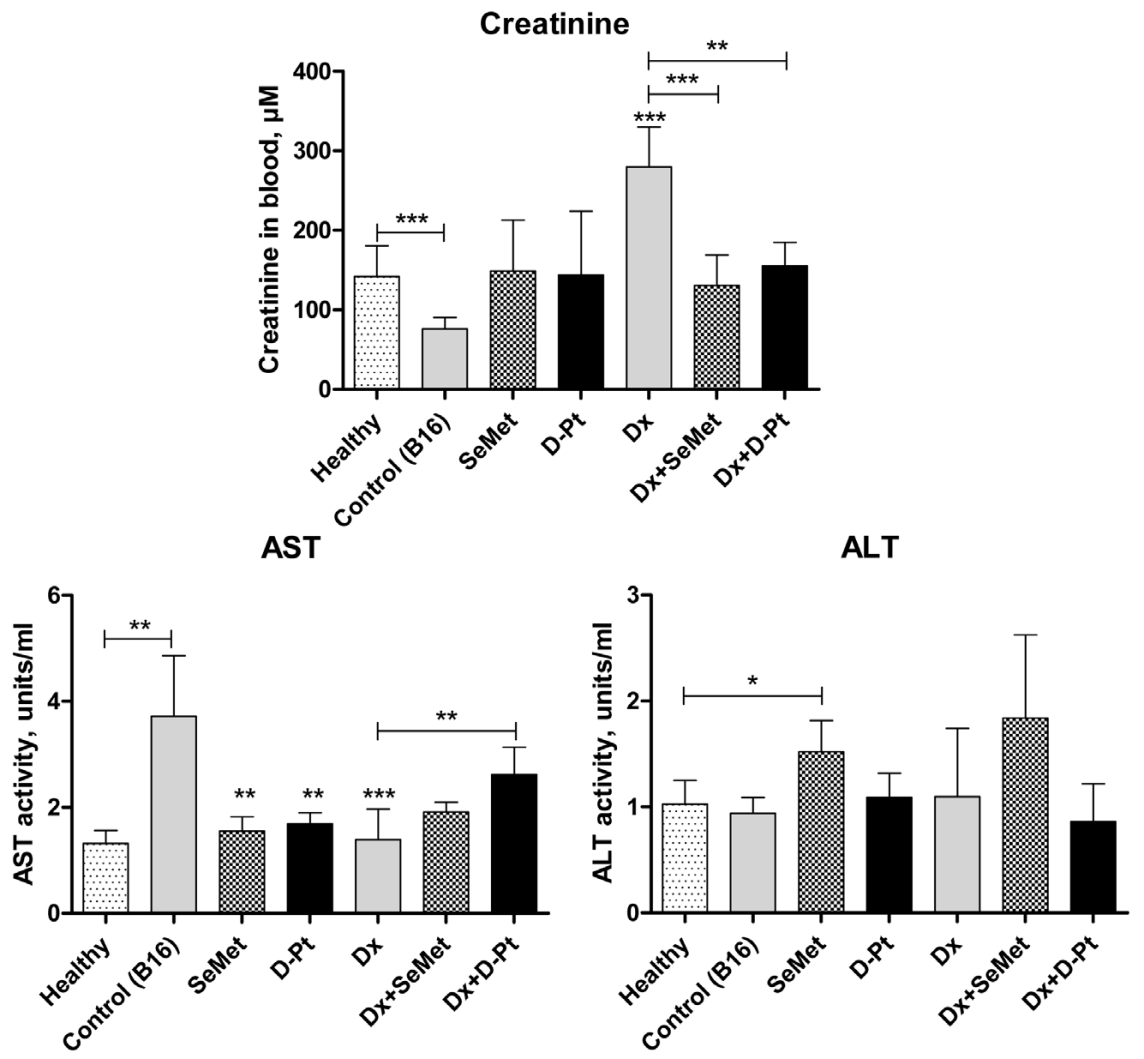
Changes in level of creatinine, activity of aspartate aminotransferase and alanine aminotransferase in B16 melanoma bearing animals treated with Dx and antioxidant compounds, at the 20^th^ day after tumor inoculation. **P < 0.01 related to control, unpaired *t* test. ***P < 0.001 related to control, unpaired *t* test.

We found that growth of B16 melanoma was accompanied by a severe cachexia revealed as a relatively weak increase in the total body weight, in contrast to big tumor volumes in control mice (Figure 6). In addition, B16 melanoma-bearing animals were characterized by twice lower level of creatinine (*P* < 0.001) compared to healthy mice (Figure 7). It is known that creatinine level in blood is tightly dependent on the fluctuations of muscle mass (20), thus, cachexia-derived loss of muscle mass might be the main reason for low creatinine found in blood of tumor-bearing animals. Dx therapy, despite inhibiting of tumor growth, was also found to be nephrotoxic, increasing 4-fold creatinine level compared to such level in B16-bearing animals and 2-fold – compared to healthy control group. It should be noted that both SeMet and D-Pt efficiently lowered creatinine level in blood of melanoma-bearing animals to the level observed in healthy animals ( ~ 140 µM). Thus, both studied antioxidants, despite their insignificant inhibitory effect on the growth of B16 melanoma, demonstrated an efficient protection against the nephrotoxic action of Dx.

B16 melanoma-bearing animals were also shown to be suffering from the tumor-induced hepatotoxicity, as revealed by a 3-fold increase in aspartate aminotransferase (AST) activity in blood serum of animals (Figure 7). Dx therapy reversed it to the values observed in healthy animals, while the combination of Dx with SeMet or D-Pt had shown the effect identical to Dx action (Figure 7). There were no significant changes found in the level of another enzyme – alanine aminotransferase, neither under tumor growth, nor under treatment with applied chemotherapies. Thus, no signs of the hepatotoxicity of Dx were observed in B16 melanoma-bearing animals suggesting that SeMet and D-Pt do not possess visible protective effects here.

**Figure 8:**
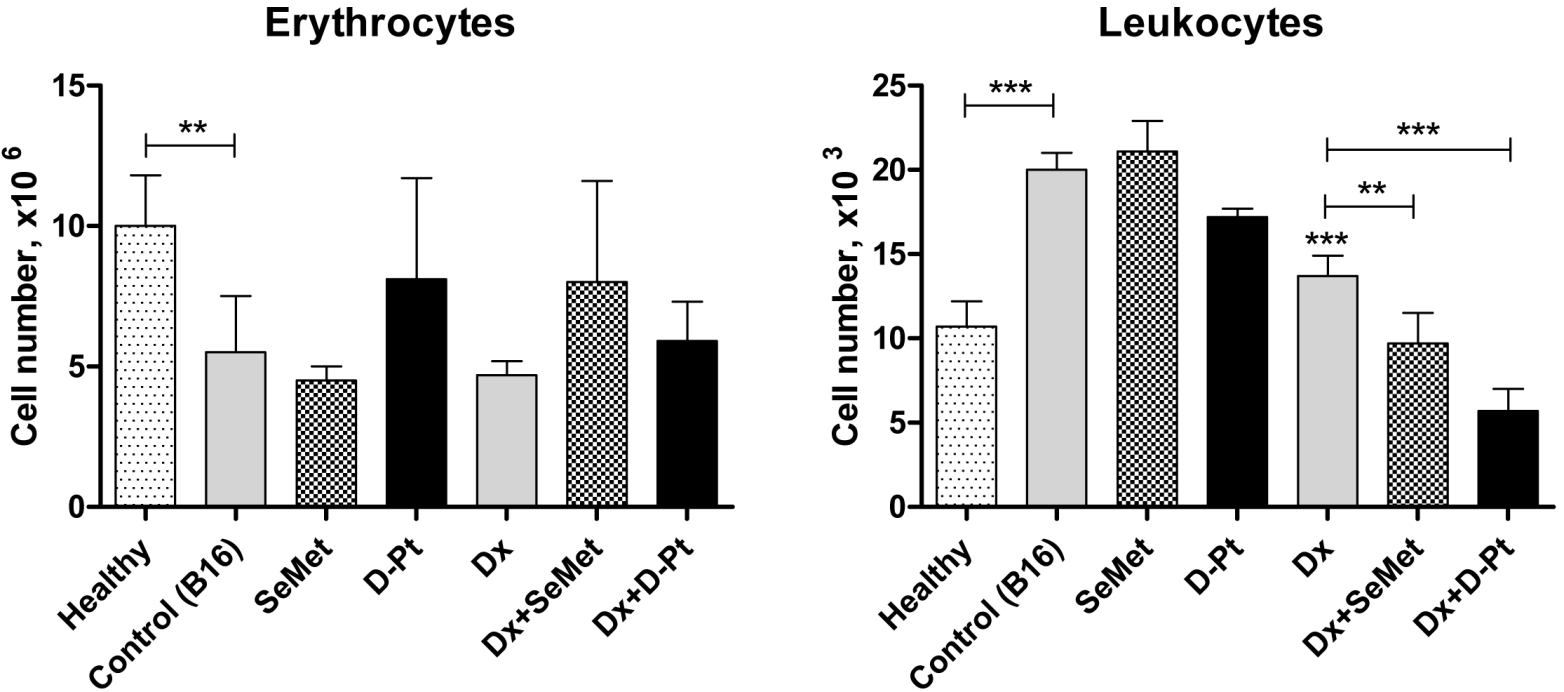
Comparison of number of erythrocytes and leukocytes in B16 melanoma-bearing animals, treated with Dx and antioxidant compounds, at the 20^th^ day after tumor inoculation. **P < 0.01 related to control, unpaired *t* test. ***P < 0.001 related to control, unpaired *t* test.

**Figure 9:**
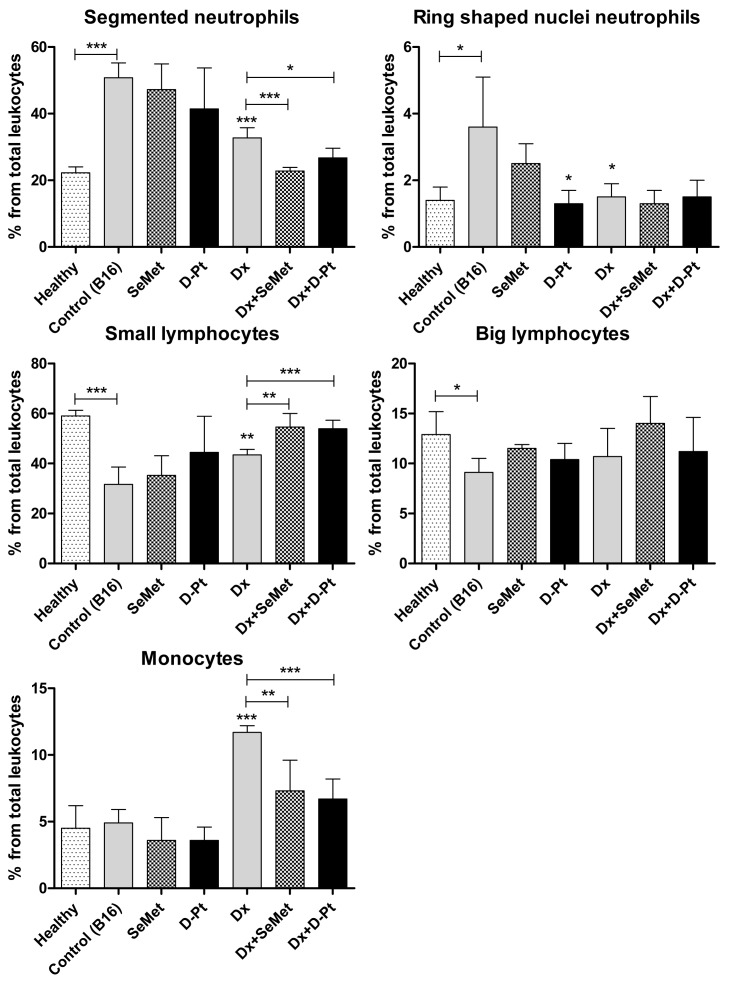
Changes in leukogram in B16 melanoma-bearing animals, treated with Dx and antioxidant compounds, at 20th day after tumor inoculation.*P < 0.05 related to control, unpaired t-test. **P < 0.01 related to control, unpaired t-test. ***P < 0.001 related to control, unpaired t-test.

Previously, we found that SeMet and D-Pt possessed strong myeloprotective activities toward Dx action in mice with NK/Ly lymphoma (11). Here, the in-depth studies of blood profile of animals with B16 melanoma treated with the same combination of drugs were performed. Blood samples were taken from tumor-bearing animals at the 10^th^ day after chemotherapy start (the 20^th^ day after tumor inoculation), and blood smears were prepared and compared with such smears, prepared from blood of healthy (control) animals (Figures 8 and 9). As one can see (Figure 8), growth of B16 melanoma is accompanied by a severe erythropenia and leukocytosis, while treatment of animals with Dx leads to further decrease in the number of erythrocytes, although it partially reverses leukocytosis (*P* < 0.001). D-Pt reversed erythropenia in tumor-bearing animals, and also partially increased their number in blood under Dx action, while SeMet had no impact here (Figure 8). Both antioxidants revealed a strong inhibitive impact on the leukocytosis, since combined treatment of mice with SeMet and Dx lowered the number of leukocytes almost to the level found in healthy animals, while a combination of Dx+D-Pt diminished this index even further – up to 60% of the control level (Figure 8).

Tumor-derived leukocytosis was characterized by two more important changes – 2-fold decrease in number of small lymphocytes with a simultaneous 3-fold increase in the level of segmented neutrophils and young neutrophils with ring-shaped nuclei (Figure 9). Treatment of animals with Dx led to partial normalization (150% of control level) of all above mentioned indices, while a combination of Dx and SeMet or, to lower impact, with D-Pt completely reversed the number of neutrophils and small lymphocytes in blood of mice to the appropriate indices found in control group of healthy animals.

Finally, Dx treatment led to a significant monocytosis in B16-melanoma bearing animals (Figure 9). Such phenomenon was observed when another experimental model, NK/Ly lymphoma, was studied (11). Co-treatment of animals with SeMet or D-Pt reversed this parameter to sub-control levels, thus, indicating their myeloprotective properties.

Summarizing, the observed reversal of leukocytosis, neutrophilia, lymphopenia and monocytosis in B16 melanoma-bearing mice under their co-treatment with Dx and antioxidants might decrease the intensity of inflammatory processes switched on by tumor growth that could provide better quality of animals’ life. This, in turn, might lead to lowering of cachexia effects in tumor-bearing animals, stabilization of their muscle mass, and normalization of creatinine levels.

## Discussion

Use of antioxidants, especially vitamin C, as a supportive therapy for treatment of nearly all diseases – starting from flu and finishing with cancer, gained an extreme popularity in the last decades. It is known that cancer patients often use without doctors control the dietary antioxidant supplements during the conventional cancer treatment in hope to palliate side effects of the chemotherapeutic drugs and, thus, to improve their health conditions (7). General idea of such a massive use of dietary supplements at cancer treatment is based on the opinion that antioxidants help to protect and repair healthy cells that are damaged by the chemotherapy via quenching free radicals whose production is induced by the anti-cancer drugs. However, there is still no high-level evidence of the benefits of the combined use of antioxidants with conventional anticancer therapies for safety of cancer patients (21,22). Moreover, it is known that cancer cells are characterized by an increased ROS level (23), and application of antioxidants might actually decrease the efficiency of chemotherapy and worsen the prognosis in cancer patient (24). Thus, more studies of molecular mechanisms of tissue-protecting action of the antioxidants have to be performed in order to reveal positive effects of their application in clinical medicine.

We have shown (10) that the organic selenium derivative – selenomethionine (SeMet) and vitamin B_5_ precursor – D-pantethine (D-Pt) are capable of diminishing several side effects of Dx action in healthy rats, namely, they abolished the oxidative stress in blood cells and protected from a decrease of CoA level in liver. We conducted studies using mice with NK/Ly lymphoma and showed that these dietary supplements, besides lowering the myelotoxicity of Dx, also lead to a boost of survival time of tumor-bearing animals (11). One of the aims of present study was to verify those data using more aggressive solid tumor model – murine B16 melanoma. We also evaluated the potential molecular mechanisms of tissue-protective action of SeMet and D-Pt.

The dissection of ROS production at a systemic cancer therapy is a critical issue, since ROS might not only be mode-of-action for the anticancer drugs, but they also might be the main cause of dose-limiting adverse effects. In that respect, the use of anthracyclines (eg, Dx) is particularly problematic. While the role of ROS in their *in vitro* and *in vivo* anticancer effects is questionable (25,26), the contribution of superoxide in cardiotoxicity is the major side effect (27).

It is believed that antioxidants protect normal cells from ROS-producing drugs by using a direct scavenging of the reactive oxygen species (28). Here we demonstrated that neither SeMet, nor D-Pt possessed such activity toward the action of Dx – a typical ROS inducing drug (3). *In vitro* studies conducted on B16 melanoma cells have revealed these cells are internally resistant to Dx action, with LC_50_ = 5 µM (drug concentration that leads to killing 50% of tumor cells), and LC_75_ dose 10 µM. In such doses, Dx caused a significant (4-5-fold) and time-dependent increase in the level of hydrogen peroxide and superoxide anions that were measured by the DCFDA and DHE assays, correspondingly (Figures 4 and 5). However, SeMet and D-Pt failed to modulate these effects of Dx at most of studied time points (3-12 h). A statistically significant decrease (30%, *P* < 0.001) of superoxide anions under SeMet addition was observed only in 24 h after the start of B16 melanoma cell treatment with high dose of Dx (10 µM). D-Pt was capable of partial decreasing hydrogen peroxide levels, but only at 24-hour cell treatment with a lower dose of Dx (5 µM). These results allow one to suggest that in the case of B16 melanoma ROS scavenging capacity of SeMet and D-Pt do not play a decisive role in modulating Dx toxicity. As mitochondria are considered to be one of the main cellular sources of ROS (19), we evaluated the impact of SeMet and D-Pt on Dx-induced mitochondrial damage using Rhodamine 123 assay. It was revealed that both dietary supplements lacked any mitochondria-protective activity toward B16 melanoma cells, and, thus, failed to protect them from Dx-induced oxidative stress.

It should be stressed that *in vitro* studies of ROS-scavenging activities of SeMet and D-Pt were done only on tumor cell line, while these compounds might differentially act toward normal cell lines, and this question needs to be further investigated. Moreover, it was known that melanoma cells are usually characterized by the elevated basal ROS content compared to normal cells (29). Thus, absence of cyto- and ROS-protective effects of SeMet and D-Pt toward cultured B16 melanoma cells under Dx action might suggest that these compounds do not interfere with Dx therapeutic action *in vivo*, which was in fact observed by us. This might be a huge benefit for using SeMet and D-Pt in clinics. Both SeMet and D-Pt gradually increased quality of life of tumor-bearing animals by lowering the nephrotoxicity and monocytosis caused by Dx, as well as by a significant boost of the immune status of tumor-bearing animals, as revealed by a decreased neutrophilia and increased level of small lymphocytes in blood. It should be stressed that most of the observed positive effects of SeMet and D-Pt were found only at their usage in a combination with Dx. Normalization of creatinine level in blood of B16 melanoma-bearing animals that was decreased in control (non-treated) group comparing to healthy animals, was the only detected positive impact of SeMet and D-Pt applied alone. Such a decrease in creatinine level in blood of tumor-bearing mice might be explained by a severe cachexia (30) that was observed as a weak increase of animal body weight, in contrast to an intensive growth of tumor volume.

A systemic inflammation and disorders of lipid metabolism are considered to be the main triggering factors at cancer cachexia (31). Therefore, one might hypothesize that a reduced manifestation of cancer cachexia in animals treated with SeMet or D-Pt is associated with their immunomodulatory action aimed at decreasing of the inflammation processes. This, in turn, could diminish muscle mass loss that is the main consequence of cachexia in tumor-bearing organism.

However, the immunomodulatory action of SeMet and D-Pt cannot explain their nephroprotective properties under Dx therapy, since it is known that Dx-induced nephrotoxicity is caused mainly by ROS produced by this drug (5). Our findings demonstrated that the molecular mechanisms of cell protection by SeMet and D-Pt against Dx action seem to be more complicated than a simple scavenging of ROS whose production is induced by Dx in damaged mitochondria. The observed phenomenon could be explained by a potential involvement of cellular glutathione system, as shown by us earlier (10,11), however, it needs further elucidation.

It should be stressed that in most animal tumor model studies, measuring of only the acute toxicity of applied drugs was possible, and the experiments were usually terminated in 30-60 days, while Dx-induced cardiotoxicity is usually observed much later, up to a year after chemotherapy start in human cancer patients (32). Thus, the performed experiments using murine B16 melanoma have limitations, since they cannot reveal the long-term outcomes of the proposed poly-chemotherapy scheme based on a combination of Dx, SeMet and/or D-Pt. This was the reason why we did not include the cardiotoxicity tests in our studies, as the analyzed periods of time – 22 days for control group and 60 days for drug-treated groups – were too short for the development of visible manifestations of heart failure in the experimental animals. For such studies, less aggressive and slowly growing solid tumor models should be used, since they could allow animal observation during a longer period of 90-120 days.

The absence of cumulative therapeutic effects of Dx+SeMet/D-Pt co-treatment, comparing to single Dx injections, might suggest a preferable use of Dx in a combination with the studied antioxidants. Normalization of blood formula and the level of red blood cells, as well as stabilization of protein metabolism (according to the creatinine level), are important factors increasing chances for a long-term survival of cancer patient due to an abolishing of negative impact of chemotherapy on the organism.

In conclusion, current study demonstrated a distinct tissue-protective activity of SeMet and D-Pt toward acute toxicity of Dx on B16 murine melanoma. As revealed by the results of our *in vitro* assays on B16 melanoma cells, such effects of SeMet and D-Pt are not connected with a direct ROS scavenging and protection of mitochondria from damage, they rather suggest other mechanisms underlying the cytoprotective action of these antioxidants toward normal cells and tissues. Further in vivo studies addressed on revealing of the molecular mechanisms of tissue-protecting activity of SeMet and D-Pt are in progress.
